# Alemtuzumab-associated diffuse alveolar damage – a case report

**DOI:** 10.1186/s12883-020-01934-7

**Published:** 2020-09-23

**Authors:** Antonios Bayas, Martina Menacher, Martin Schwaiblmair, Bruno Märkl, Markus Naumann

**Affiliations:** 1 Neurology and Clinical Neurophysiology, University Hospital of Augsburg, Stenglinstrasse 2, 86156 Augsburg, Germany; 2 Internal Medicine I-Cardiology, University Hospital of Augsburg, Stenglinstrasse 2, 86156 Augsburg, Germany; 3 General Pathology and Molecular Diagnostics, Medical Faculty, University of Augsburg, Stenglinstrasse 2, 86156 Augsburg, Germany

**Keywords:** Multiple sclerosis, Alemtuzumab, Diffuse alveolar damage, Case report

## Abstract

**Background:**

Identifying causes of alemtuzumab induced respiratory symptoms in Multiple Sclerosis (MS) patients is crucial.

**Case presentation:**

We report a case of diffuse alveolar damage (DAD) in a patient with MS after the first course of alemtuzumab treatment. A 42-year-old female developed progressive non-productive cough and exertional dyspnea 2 months after alemtuzumab treatment. DAD was diagnosed histopathologically by lung biopsy. The patient recovered completely, alemtuzumab was not continued.

**Conclusions:**

Our case highlights another pathomechanism for non-infective lung-disorders in alemtuzumab treated MS patients. DAD is a potential, albeit rare side effect of alemtuzumab, broadening the spectrum of non-infective lung disorders that should be considered in the diagnostic work-up.

## Background

Alemtuzumab is a humanized monoclonal antibody, which targets the surface molecule CD52 on immune cells (T- and B-cells, monocytes, dendritic cells and thymocytes) and leads to a rapid and significant depletion of those cell types [1]. Well-known side effects include an increased infection rate including respiratory tract infections and secondary autoimmune reactions mainly consisting of thyroid disorders, immune thrombocytopenia, anti-glomerular basement membrane (GBM) disease and membranous glomerulonephritis, which may occur after years of first treatment. Since its approval by the European Medicines Agency (EMA) in 2013, non-infective respiratory tract side effects causing dyspnea have been reported in patients treated with alemtuzumab including diffuse alveolar hemorrhage [2], pneumonitis [3], anti-GBM antibody disease [4] and acute respiratory distress syndrome (ARDS) [5]. Five cases of diffuse alveolar damage (DAD) associated with alemtuzumab have been reported to VigiBase©, the World Health Organization’s international database for suspected adverse drug reactions [6], without providing further details on treated disorders or other potentially confounding factors. Our report describes a patient with DAD after alemtuzumab treatment for MS. Compared to cases of alemtuzumab associated DAD published so far [6], here we present detailed clinical data and results of apparative diagnostics, furthermore histological findings that have so far not been reported in ARDS associated with alemtuzumab treatment [5].

## Case presentation

A 42-year-old, non-smoking female with highly active relapsing remitting multiple sclerosis (MS) and no concomitant diseases was first diagnosed in 2008. Interferon beta (IFNB)-1a intramuscularly, injected 03/2008, was stopped due to a suspected allergic reaction, intradermal testing for IFNB-1a and -1b could not exclude an allergic reaction. Glatiramer acetate had to be withdrawn after 4 months (07/2008–11/2008) for elevated liver enzymes. Due to high disease activity natalizumab was initiated in 2009 and stopped in 2012 because of JC-virus antibody positivity, hereafter the patient refused further immunotherapies. After a severe relapse resolving only after plasma exchange (PLEX), treatment with fingolimod was initiated, but stopped after 4 months due to another severe relapse requiring treatment with glucocorticosteroids and again PLEX. After 2 further relapses and magnetic resonance imaging (MRI) activity in 2013, treatment with alemtuzumab was decided.

**Fig. 1:**
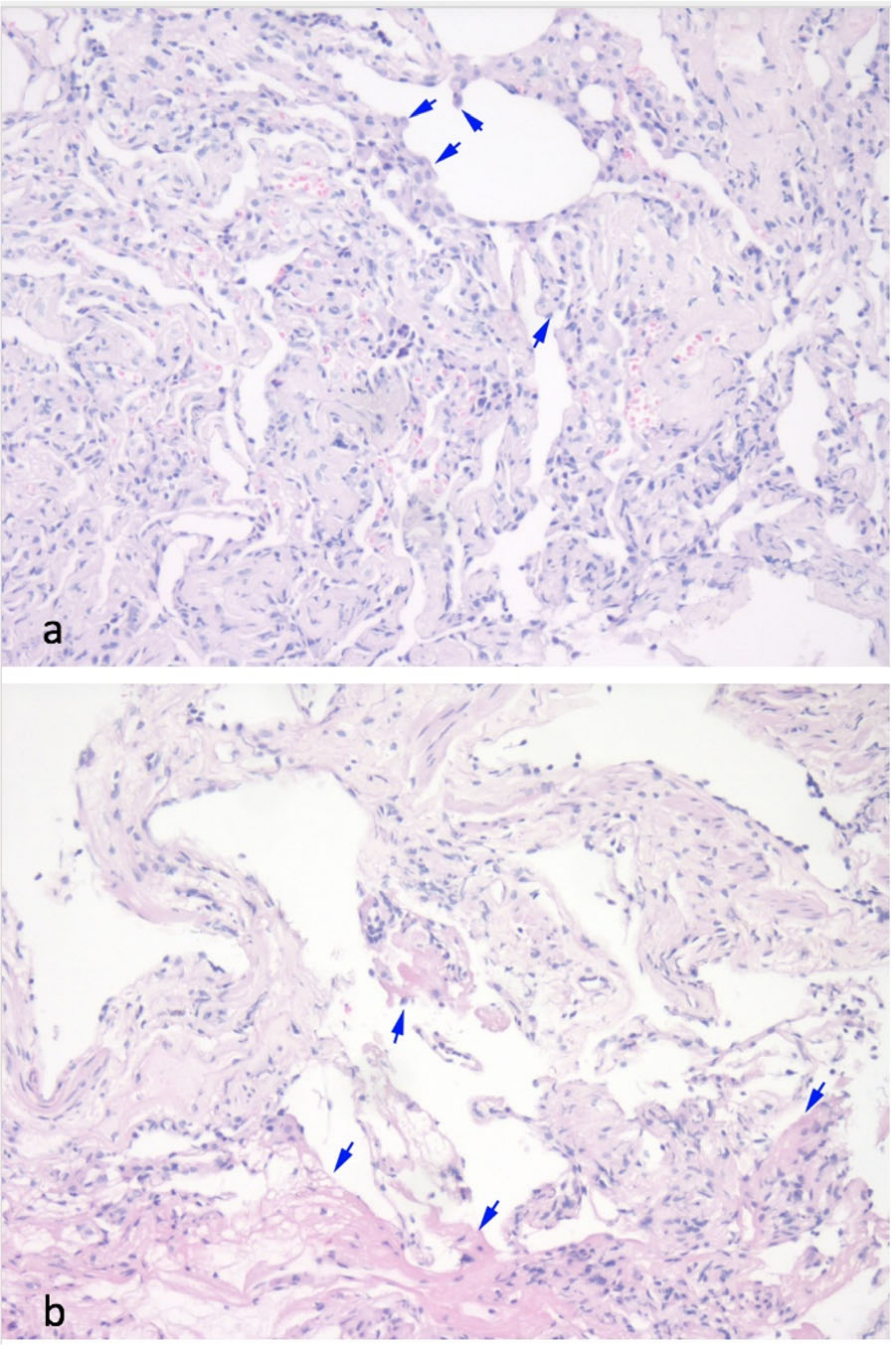
Lung biopsy, hematoxylin and eosin stain: **a** arrows indicate activated pneumocytes, **b** arrows indicate residual hyaline membranes

In January 2014 alemtuzumab (12 mg daily) was applied over 5 days, methylprednisolone 1 g, dimetindene, ranitidine, acyclovir and ibuprofen were given as concomitant medication. Except of fatigue and a mild bradycardia, infusions were well-tolerated. After 2 months, progressive non-productive cough, exertional dyspnea and general exhaustion developed. C-reactive protein and leucocytes were normal. Computed tomography- (CT-) scan of the lung showed atelectasis in both lower lobes without evidence of atypical pneumonia. Lung function tests showed a high-grade restriction in diffusion capacity (diffusing capacity of the lung for carbon monoxide (D_LCO_) 45% of normal) as well as a respiratory alkalosis (pH 7.46, pCO_2_ 34.3 mmHg), a respiratory partial insufficiency in ergospirometry (decrease of pO_2_ from 89 mmHg to 78 mmHg and increase of lactate from 1.0 to 5.5 mmol/l during exercise with 150 watt) and a low-grade restrictive lung disease (vital capacity (VC): 2.75 l, reference: ≥ 3.70 l) without obstructive impairment (FEV_1_ (forced expiratory volume in 1 s)/VC: 88.7%, reference: ≥ 80.9%). Bronchoalveolar lavage revealed a moderately active inflammation and a reduced CD4/CD8-ratio (0,67; reference ≈ 2) consistent with an extrinsic allergic alveolitis or a damage of pneumocytes due to drug toxicity. However, an extrinsic allergic alveolitis seemed unlikely because of negative specific IgG antibodies. The Prick test and Methacholin provocation test, suggesting an allergic reaction or a hyperreagibility of the bronchial system, were negative. Histopathologically, hyperplasia of alveolar pneumocytes and remnants of hyaline membranes consistent with a diffuse alveolar damage were found in a transbronchial lung biopsy (Fig. 1). There was no evidence for an infectious agent. Renal function, urine test and anti-GBM antibodies indicating an alemtuzumab-associated Goodpasture syndrome, were unremarkable. Platelets and thyroid function were normal, thyroid antibodies were not determined.

Based on these results, a diffuse alveolar damage (DAD) related to alemtuzumab drug toxicity was diagnosed. Respiratory symptoms regressed over few months, no corticosteroids were given. Diffusion capacity in December 2014 and December 2015 had constantly improved (single-breath-methode, D_LCO_ 71,5 and 81% respectively of normal). Blood gas analysis and lung function tests were normal lately.

**Fig. 2:**
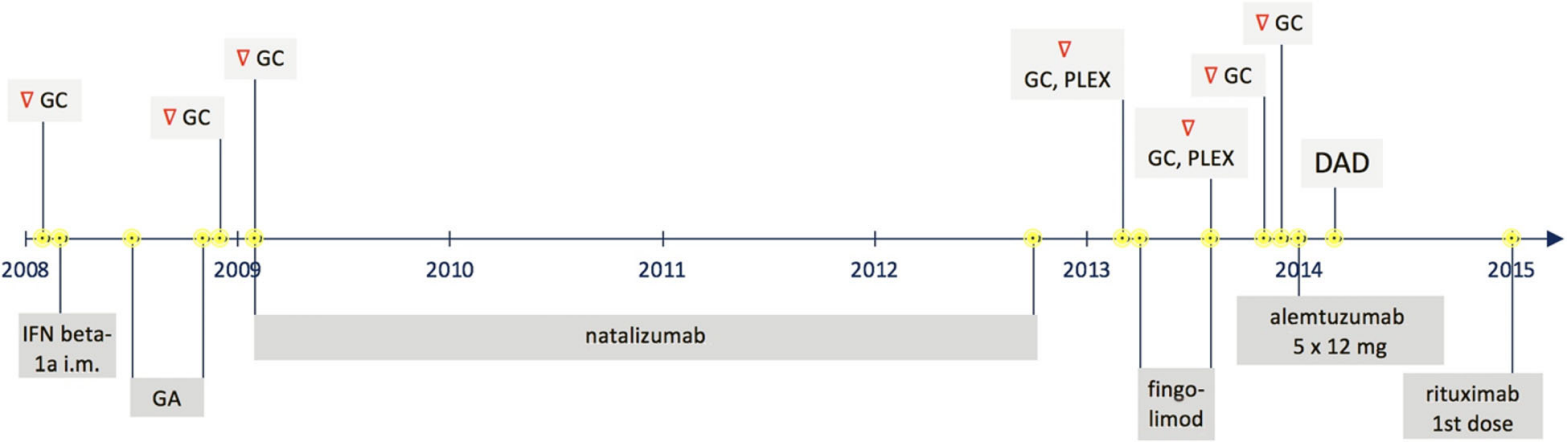
Disease course and applied treatments. IFN = interferon; GA = glatiramer acetate; ∇ = relapse; GC = glucocorticosteroids; PLEX = plasma exchange; DAD = diffuse alveolar damage

Due to the increased risk for recurrent toxicity, alemtuzumab was not continued and treatment switched to rituximab (off-label) in January 2015. MS clinical course and MRI controls have been stable since 2014. The disease course and treatments are shown in Fig. 2. Written informed consent for patient information and images to be published was provided by the patient.

## Discussion and conclusions

Over the last years, reports of serious adverse events also affecting the lungs have prompted the EMA to amend the label for alemtuzumab [7]. More recently, further cases of treatment-induced lung injury have broadened the spectrum of non-infective respiratory disorders after alemtuzumab treatment in MS [5]. To our knowledge, our case is the first report of DAD in MS after alemtuzumab treatment examined histologically. ARDS as a clinical correlate of DAD after alemtuzumab treatment in MS has recently been published, but lung biopsy is not reported [5]. DAD is characterized by dyspnea and dry cough progressing over a period of days or weeks. High-resolution CT typically shows a combination of ground-glass opacity and consolidation involving predominantly dependent regions or distributed more randomly throughout both lungs [8]. DAD can be induced by infections, inhalational injuries, connective tissue diseases and also drugs [9]. It is the most commonly reported histologic manifestation of drug toxicity usually developing after a few weeks or months [8]. The mortality rate in DAD has been reported to be high. In a series of 58 patients with DAD diagnosed by surgical lung biopsy over a 7-year period, 60% were immunocompromised. In this study, the most common cause of DAD were infections (22%), drugs were found to be causal in 10% of cases. The overall hospital mortality rate associated with DAD was 53%, in the group of drug induced DAD 1 of 6 patients died [9]. In our case, respiratory symptoms occurred 2 months after the alemtuzumab cycle, according to the latency known from other drugs. Fortunately, the patient reported gradually recovered over months without subjective residual symptoms. Apart from atelectasis, the initial CT scan revealed no abnormal findings indicating a milder lung involvement and possibly explaining a less severe disease course.

Various pathological phenomena underlying DAD have been reported, among them endothelial and alveolar cell injury leading to fluid and cellular exsudation with hyaline membranes and edema [10]. The mechanism underlying alemtuzumab induced DAD is unknown. In diffuse alveolar hemorrhage after alemtuzumab treatment, it has been speculated that alemtuzumab induced effector mechanisms lead to acute inflammation that may damage membranes and cells not expressing CD52 [2].

Our report shows that DAD is a potential, albeit rare side effect of alemtuzumab, broadening the spectrum of non-infective lung disorders that should be considered in the diagnostic work-up.

## Data Availability

All data relevant for the case report have been presented in the manuscript. Further, potentially identifying or confidential patient data will not be shared.

## Abbreviations

MS: Multiple sclerosis
DAD: Diffuse alveolar damage
GBM: Anti-glomerular basement membrane
EMA: European Medicines Agency
ARDS: Acute respiratory distress syndrome
IFNB: Interferon beta
PLEX: Plasma exchange
MRI: Magnetic Resonance Imaging
CT: Computed tomography
VC: Vital capacity
